# Comparing multifocal pupillographic objective perimetry (mfPOP) and multifocal visual evoked potentials (mfVEP) in retinal diseases

**DOI:** 10.1038/srep45847

**Published:** 2017-04-03

**Authors:** Faran Sabeti, Andrew C. James, Corinne F. Carle, Rohan W. Essex, Andrew Bell, Ted Maddess

**Affiliations:** 1 Eccles Institute of Neuroscience, John Curtin School of Medical Research, Australian National University, Canberra, Australia; 2 Department of Ophthalmology, The Canberra Hospital, Canberra, Australia; 3 Medical School, Australian National University, Canberra, Australia.

## Abstract

Multifocal pupillographic objective perimetry (mfPOP) shows regions of slight hypersensitivity away from retinal regions damaged by diabetes or age-related macular degeneration (AMD). This study examines if such results also appear in multifocal visual evoked potentials (mfVEPs) recorded on the same day in the same patients. The pupil control system receives input from the extra-striate cortex, so we also examined evidence for such input. We recruited subjects with early type 2 diabetes (T2D) with no retinopathy, and patients with unilateral exudative AMD. Population average responses of the diabetes patients, and the normal fellow eyes of AMD patients, showed multiple regions of significant hypersensitivity (p < 0.05) on both mfPOP and mfVEPs. For mfVEPs the occipital electrodes showed fewer hypersensitive regions than the surrounding electrodes. More advanced AMD showed regions of suppression becoming centrally concentrated in the exudative AMD areas. Thus, mfVEP electrodes biased towards extra-striate cortical responses (surround electrodes) appeared to show similar hypersensitive visual field locations to mfPOP in early stage diabetic and AMD damage. Our findings suggest that hypersensitive regions may be a potential biomarker for future development of AMD or non-proliferative diabetic retinopathy, and may be more informative than visual acuity which remains largely undisturbed during early disease.

Since 2009 our group has published several reports using *multifocal pupillographic objective perimetry* (mfPOP)^1^,^2^,^3^,^4^,^5^,^6^,^7^,^8^,^9^,^10^,^11^,^12^. The method was pioneered by others^13^,^14^. Like multifocal electroretinograms (mfERGs)^15^,^16^,^17^, and multifocal visual evoked potentials (mfVEPs)^18^,^19^,^20^ mfPOP concurrently presents many visual stimuli, but the measured responses are video records of changes in the diameter of the pupils, obviating the need for electrodes. With current mfPOP methods both eyes are tested at the same time. Test duration is under 7 minutes and the tests are tolerant of blinks and fixation losses. Relative changes in pupil size are used to minimise the effect reduced pupil size in older persons. The input to the irises is from the pretectal olivary nucleus (PON), which receives both direct afferent input from the eyes, and from the extra-striate visual cortex^21^. Indeed at least 8 extra-striate areas provide input to the pupils^21^. Best known of these inputs is the strong driver of pupil responses to red-green equiluminant stimuli, which can be reversibly modulated from macaque cortex^22^, or blocked by similar cortical lesions in humans^23^. The subjects with lesions also show red-green achromatopsia. The mfPOP method presents sparse trains of transiently presented stimuli, and we have shown that these significantly drive the cortical pathway to the PON^11^,^24^.

Originally developed for glaucoma the mfPOP method has shown surprisingly high sensitivity and specificity for diagnosing both very early stage macular degeneration, and diabetic eye damage even before the appearance of vascular changes to the retina (area under Receiver Operating Characteristic (ROC) plots > 0.9). The most successful mfPOP variant for retinal disease tests 44 regions of the macular field of each eye concurrently^5^,^6^,^9^,^12^. The most powerful diagnostic measure for the earliest stages of AMD and diabetic eye disease is asymmetries in sensitivity between anatomically equivalent visual field regions of the two eyes^2^,^5^,^6^,^9^,^12^. This is driven by a combination of visual field regions showing both sensitivity reductions and moderate sensitivity increases, and is assisted by the two eyes being tested simultaneously, providing a within subject control. In the earliest stages of diabetes the localised sensitivity increases measured by mfPOP can provide better sensitivity and specificity than sensitivity losses^25^.

Figure 1 illustrates the average difference from normal eyes reproduced from three published mfPOP studies of retinal diseases^2^,^5^,^6^. In each panel the grey background represents no difference from normal sensitivity, darker tones indicate reduced sensitivity, lighter hypersensitivity. Even in these averages across up to 50 eyes many of the hypersensitive regions are significantly different than normal. Figure 1A is from a study of persons commencing anti-VEGF treatment for their Wet AMD. That study showed that eyes showing hypersensitive regions responded significantly better to treatment^6^. Figure 1B is from a study of 25 type 2 diabetics and age-matched controls. Here asymmetry between individual left and right eyes, in part driven by regions of hypersensitivity, drove AUC values as high as 95%. Figure 1C,D are from a study of eyes with a range of severities of AMD from early to late stage Exudative (Wet) AMD. The two figures illustrate the general findings of our mfPOP studies of retinal disease^2^,^5^,^6^,^9^,^12^, (1) earlier stage disease displays a mix of hypo- and hyper-sensitive regions, (2) these later give way to concentrated sensitivity loss, (3) eyes with no hypersensitive regions respond poorly to treatment^6^. Mildly hypersensitive regions appear to be more common when AMD patients are tested with mfPOP at photopic rather than at scotopic levels^7^.

Localised sensitivity increases have not been reported in studies using standard automated perimetry of retinal disease. This is not surprising because perimeters do not flag significantly elevated sensitivity, only depressed sensitivity relative to normal control data. Average perimetric sensitivities of +3 to +4 decibels (Mean Defects) have, however, been reported in diabetic patients with no retinopathy using Short Wavelength Automated Perimetry (SWAP)^26^. All this suggests that the hypersensitivities seen with mfPOP are derived from physiological processes related to early-stage retinal disease, but what is their source? In particular do they represent up-regulation of compensatory mechanisms in the eye that fail in later disease stages, or do cortical factors play a role, or both?

We recorded mfVEPs in two mfPOP studies of retinal disease: the study of Fig. 1B on Type 2 diabetes (T2D) with no retinopathy^2^, and the study of Fig. 1C,D on persons with unilateral exudative AMD^5^. We used the mfPOP platform to stimulate both eyes simultaneously with 84 stimulus regions/eye while recording VEPs with 64 EEG electrodes. The mfPOP and mfVEP tests were done on the same day for every subject. The mfVEP study of AMD has been partially published^20^, but in that publication we only examined responses from the few electrodes placed directly above the occipital cortex. The mfVEP study of T2D has not been published. Previous reports of mfVEP responses from occipital electrodes in patients with T2D showed only significant implicit time delays in the absence of retinopathy; however those delays correlated poorly with regions of loss on mfERG^27^. Using mfPOP stimuli we have recently confirmed earlier literature^21^,^28^ showing that the pupillary system can be substantially driven by the visual cortex^29^, especially when using the transient stimuli of mfPOP^24^. Given those findings, and the brain lesion studies indicting substantial involvement of the extra-striate cortex in pupillary responses of primates and humans^22^,^30^, we here compare the mfVEP responses from electrodes covering the occipital cortex, and surrounding electrodes that are more biased to extra-striate cortex, for any sign of the hypersensitivities in the subjects from those two studies.

## Methods

### Patients

Data from two separate studies are presented here. In Study 1^5^ we recruited nineteen patients from The Canberra Hospital Ophthalmology department with active unilateral choroidal neovascularization (mean age = 77.7 ± 5.4 years; 49% female) secondary to age-related macular degeneration (AMD). The fellow eye presented with normal (*n* = 4) or non-exudative AMD (*n* = 15). We recruited twenty-eight age-matched control participants (mean age = 70.8 ± 6.1 years).

Study 2^2^ included twenty-three T2D (mean age = 58.0 ± 6.9 years; 62% female) from cooperating General Practices. A retinal specialist (RWE) graded the diabetic retinopathy in the fundus photographs and was masked to mfPOP and mfVEP results. In the diabetes group twenty-one subjects were classified as having no retinopathy and two subjects presented with mild non-proliferative diabetic retinopathy. Twenty-three age and sex matched control subjects (mean age = 59.7 ± 5.6 years) were included in this study.

All participants’ diagnoses were confirmed using best corrected visual acuity (BCVA), fundus biomicroscopy, intraocular pressure, Frequency Doubling Perimetry C-20 fields and optical coherence tomography (OCT; Zess Cirrus: Carl Zeiss Meditec Inc. Dublin, California, USA). Subjects were excluded if they had any evidence of other ocular or neurologic disease or significant ametropia (≥ ± 6D sphere; >2 D cylinder). All data was collected at one visit with subjects completing mfPOP followed by mfVEP tests on the same day. Procedures complied with the Declaration of Helsinki and all participants gave informed written consent. The study was approved by the human research ethics committees of the Australian National University and The Canberra Hospital.

### mfVEP stimuli and recording

Subjects wore a head cap holding 65 electrodes in a 10–10 layout. Electrode guides were pre-filled with conductance gel (Spectra 350 Gel, Parker Laboratories Inc., Fairfield, New Jersey). We used pin-type active Ag-AgCl electrodes and ground was positioned on the right earlobe (Actiview, Biosemi Ltd, Amsterdam). Common mode sense (CMS) and driven right leg (DRL) electrodes were placed at Fz and Fpz, respectively. Signals were amplified 50,000 times, low-pass filtered at 53 Hz, and sampled at 240 samples/s (4 per video frame). The 24-bit recordings were DC-coupled allowing any reference to be computed after recording. Mean reference was used for analysis and recorded signals were digitally filtered with a bandpass of 1–20 Hz.

The nuCoria Field Analyser is stereoscopic system that allows for testing to be performed binocularly (dichoptically) at 60 video frames/s (e.g. ref. 10). The independent stimuli were presented concurrently to each eye on separate LCD monitors on the same nuCoria Field Analyser that was used for mfPOP testing. The stimulus array had an 84 element cortically scaled^18^ dartboard layout for each eye with a visual field diameter of 45 deg (Fig. 2). Stimuli were temporally modulated to deliver sparse, pseudorandom, contrast reversing stimuli with a mean frequency of 2 stimuli/s/region. These sparse stimuli create responses about 15 times larger than conventional densely flickering mfVEP stimuli^31^,^32^, with commensurate improvements in diagnostic power^19^,^20^. The resulting signal to noise ratios are improved by about square root 15 allowing for shorter duration experiments. Recording duration was 240 seconds divided into eight segments of 30 s, and subjects could rest between segments. Details of the exact stimuli have been given elsewhere^20^. Vision of the participants was corrected with trial lenses for their distance ametropia to within ± 1.5 dioptres.

### Analysis

Data analyses were completed with MATLAB (2016b, The MathWorks, Natick, MA). The combination of 84 stimulus regions/eye and 64 electrodes produced 10,752 (84 x 64 x 2) responses/subject. Our aim was to investigate differences the response profiles from the electrodes recording the occipital pole and those obtained from the surrounding regions (Fig. 3). The method for estimating the mean response to stimuli delivered to each stimulus region has been described in detail elsewhere and utilised a multiple linear regression method^31^,^32^. We averaged the response waveforms collected from 9 channels from the occipital lobe (P_1_, P_z_, P_2_, PO_3_, PO_z_, PO_4_, O_1_, O_z_, O_2_) and 8 surrounding channels (P_3_, P_5_, P_7_, P_4_, P_6_, P_8_, PO_7_, PO_8_). In addition, we quantified deviations from normative data for response amplitude and delay at each visual field stimulus region (Fig. 4). It is well established by combined fMRI and VEP methods that PO3, PO4 and nearby electrodes isolate striate cortical responses, while P7, P8, PO7, PO8 and their neighbours pick up human extra-striate cortical areas 18,19^33^,^34^ (see Discussion).

## Results

Table 1 presents the pertinent demographic data. Subjects within the healthy, AMD and DR-only groups were not significantly different in age and sex distribution.

Table 2 presents mean response amplitude differences between occipital and surround electrodes that showed significantly increased responses for both T2D (0.272 dB, p < 0.0001) and normal (0.532 dB, p < 0.003) and exudative AMD (0.341 dB, p < 0.006) patients. On average, early AMD showed reduced responses but did not reach significance (−0.109 dB, p = 0.181). Like the T2D patients with normal fundus appearance the eyes of AMD patients with normal appearance also showed significantly larger responses on the surround electrodes (0.532 dB, p = 0.0003).

**Figure 1:**
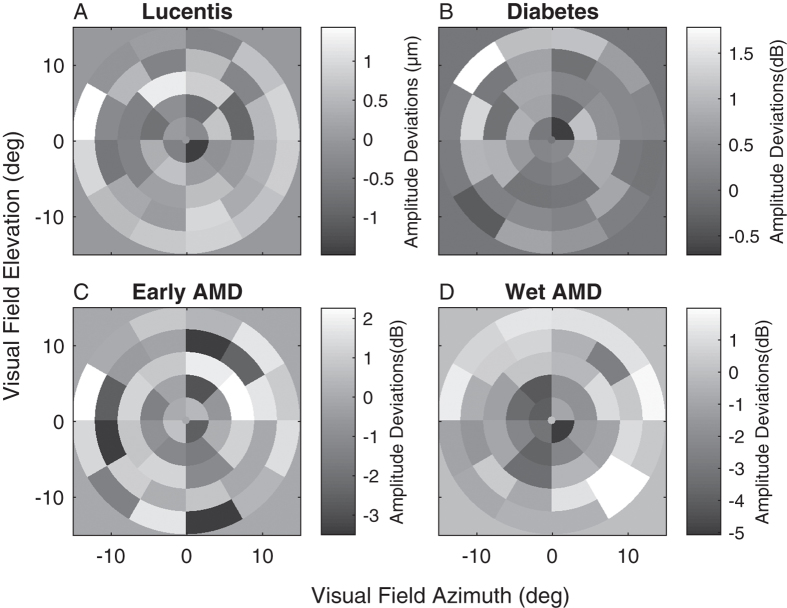
Mean deviations reproduced from 3 published mfPOP studies of retinal disease. In each case the grey-level plots indicate the average results of up to 50 left and right eyes of patients showing the average difference from normative data at each visual field location. In each panel the background grey indicates no difference from normal, darker tones indicate loss of sensitivity, lighter: hypersensitivity. (**A**) Is from eyes from a study in which persons commenced ranibizumab (Lucentis) treatment for the first time in one eye (N = 20). Individual eyes with hypersensitive regions showed significantly better reduction in retinal thickness (P < 0.0005)^6^. (**B**) Mean deviations for 50 eyes of T2D patients with up to 25 years of disease but no vascular changes. Asymmetry between eyes yielded AUC values up to 0.94^2^. (**C**) Mean deviations from normal data for 18 eyes with small to moderate drusen. (**D**) Mean deviations from normal data for 20 eyes with exudative (Wet) AMD. C and D are from the same study.^12^ In all cases data from right eyes was flipped left to right before averaging. Thus all data is presented as for left eyes, with the temporal field on the left side of each panel. All averaged field data in the paper are presented in the same way. The stimulus array of B had twice the diameter of the other stimuli, extending to ± 30 degrees eccentricity. The response units shown are those used in the original reports.

Figure 4 illustrates the average peak RMS value for each occipital electrode for controls in the T2D study (Fig. 4A) and AMD study (Fig. 4B). The mfVEP amplitudes were very similar across both groups. Smaller responses in the superior visual field are typical for VEPs. As in Fig. 1 all plots of mfVEP field data from right eyes were flipped left to right before averaging. Hence all data is presented as for left eyes with the temporal field on the left side.

Figure 5 shows the mean waveforms for controls (thick black waveforms) and patients with T2D (grey) computed across eyes, subjects, and visual field regions for both the occipital (Fig. 5A) and surrounding (Fig. 5B) electrodes from the T2D study. On average surround electrode T2D responses were larger than control subjects by 0.272 dB (p < 0.0001).

**Figure 2:**
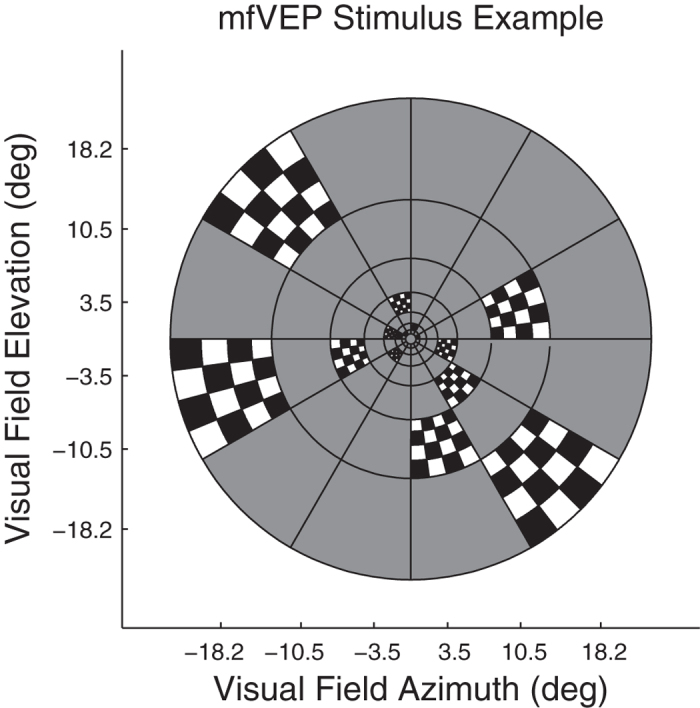
The mfVEP stimuli presented 84 M-scaled stimuli to each eye concurrently using the mfPOP dichoptic system. The figure illustrates the sparse stimuli wherein transient (33 ms) checkerboard stimuli are presented in one of two alternative contrast formats against a mean luminance grey background. The two contrasts can be appreciated by following the check colouring around each ring. The thin black lines were not present and are shown here only to illustrate where checkerboards could appear. The central 1 degree contained a red fixation cross. Recording run duration was 240 seconds divided into eight segments of 30 s.

The human striate cortex is located in the calcarine sulcus causing inverted VEP waveforms for stimuli presented above and below the horizontal meridian of the visual field (reviewed^33^,^35^). This is not true of the extra-striate cortex and response inversion, or lack of it, has been used as a marker for striate vs. extra-striate activity^33^,^35^. We therefore inspected the mean mfVEP waveforms for all field locations obtained from the occipital and surround electrodes (Fig. 6). The left and right half-rows of Fig. 6 represent the left and right radial spokes of the visual stimuli (Fig. 2)and thus model the responses projected onto the cortical sheet as we have described in detail^20^,^32^. The central two rows, representing the spokes of stimuli at polar angles ± 15 degrees off the horizontal meridian best illustrate the point. The waveforms from occipital electrodes (Fig. 6A) show clear inversion about the meridian. For surround electrodes (Fig. 6B) there is a mixture centrally, and a general lack of inversion peripherally.

Figure 7 shows mean T2D deviations from normative data (Fig. 4) computed across the occipital electrodes (Fig. 7A,B) and surrounding electrodes (Fig. 7C,D). T2D amplitude deviations were often larger than controls with no regions found to be reduced for both occipital and surrounding electrodes. The deviations are computed in a linear model with effects for region by disease, corrected for sex and age. A larger number of significantly elevated deviations (p < 0.05) were found in the visual field when surrounding electrodes were examined (Fig. 7B,D).

Average AMD Deviations from the normative data is illustrated in Fig. 8 for both Occipital (Fig. 8A to C) or surrounding (Fig. 8D to E) electrodes for three severities of AMD. Figure 8A and D show amplitude deviations for putatively normal fellow eyes of subjects who have unilateral exudative AMD in the alternate eye. The middle row shows response amplitude deviations for eyes with early AMD characterized by large drusen (>125 μm) and pigmentary changes. Figure 8C and E show amplitude deviations for exudative AMD. Similar to Fig. 7B,D, Fig. 9 presents regional amplitude deviations for the AMD patients as colour-coded probability maps, white for significant hypersensitivity, black for significant sensitivity loss (both p < 0.05). The mfVEP amplitude abnormalities present as elevated responses in the normal retina of fellow eyes of unilateral AMD subjects (Fig. 9A and D). Response amplitudes become depressed with progression to early AMD and are biased towards greater loss centrally with further progression to advanced AMD (Fig. 9C and F). Isolating the surrounding electrodes showed an increased number of elevated response amplitudes in the putatively normal eyes of AMD subjects (cf. Fig. 9A,D). Response delays were less informative and differences between occipital and surround delay deviations did not reach significance for either early AMD (0.49 ms, p = 0.15) or NPDR (0.32 ms, p = 0.13).

## Discussion

One important outcome was the agreement in functional changes observed for retinal disorders between mfPOP and mfVEP tests done on the same day. Functional impairment in patients with T2D with no retinopathy (Figs 5,7) and putatively normal fellow eyes of exudative AMD patients (Fig. 9A,D) showed evidence of larger mfVEP responses compared to controls. For both diseases hypersensitivity was more commonly reported by the surround electrodes that are likely biased to responses of the extra-striate cortex (Fig. 6). These results were also in accord with regions of hypersensitivity observed in the same subjects tested with mfPOP on the same day (Fig. 1B,C,D). Anatomically the pupillary system receives significant input from the extra-striate cortex^21^, and we have demonstrated cortical input to the responses obtained from the types of transient mfPOP stimuli used here^24^,^29^. Thus it seems plausible that observed hypersensitivities in mfPOP derive from extra-striate input.

**Figure 3:**
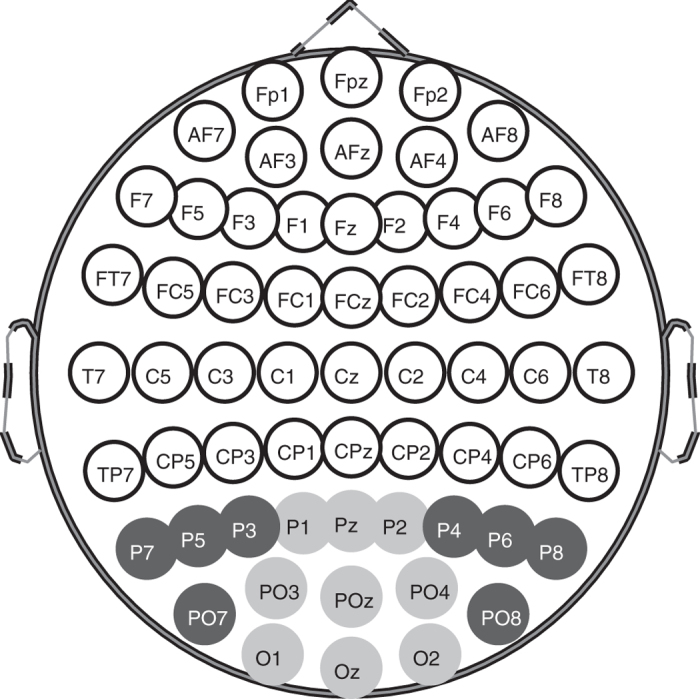
The EEG electrode array was based on a standard 10–10 layout. Responses in this study were the root mean square of response waveforms across two sets of electrodes, for each of the 84 stimulus locations. The first electrode set consisted of the occipital pole electrodes shown in light grey and also Iz, PO1 and PO3, which are not shown to avoid clutter. PO1 and PO3 are additional to the 10–10 array and are between PO3 and Oz and PO4 and Oz, giving extra weight to the striate visual cortex. The second set comprised the surrounding electrodes shown here in dark grey, and also contained P9 and P10 which are not shown.

The two most relevant investigations of sources of focal and multifocal VEP responses are those of di Russo *et al*.^33^ and Capilla *et al*.^34^. Di Russo *et al*.^33^ tested with a focal pattern-onset stimulus presented to each of the four quadrants of the visual field. Pattern onset has characteristic C1 and C2/P1 components. They reviewed the 22 papers written before 2002 that had concluded that C2/P1 were of extra-striate cortical origin (see their Table 1). Di Russo *et al*.^33^ used a 10–10 electrode array (e.g. Fig. 3) and their source analysis indicated that that electrodes P7, P8, PO7 and PO8 gave maximum expression to C2/P1. Both our mfPOP and mfVEP methods present onset stimuli. Capilla *et al*.^34^ used a 60 region, M-scaled, mfVEP stimulus presented within 22° of fixation and a 10–10 EEG array. Their stimuli were relatively slow pseudo-randomly contrast reversing stimuli. Their source analysis indicated extra-striate sources had the largest expression at PO7 and PO8. We used 84 M-scaled regions within 22.5° (Fig. 2), and had extra electrodes from the 10–5 pattern added to our 10–10 array. Another mfVEP study by Fortune and Hood^35^ is also highly relevant. They varied the reversal rate of their mfVEP stimuli from fast to slow. They did not use EEG recording but presented evidence that, like slow onset stimuli, slower reversal mfVEP stimuli had greater contributions from extra-striate sources: principally the non-reversal of waveforms for the superior vs. inferior visual fields as in Fig. 6.^35^ Taken together this information suggests that our choice of surround electrodes was reasonable.

The difference in mean response amplitudes between occipital and surround electrodes was 0.27 dB and 0.53 dB (p < 0.0003) in retinopathy-free eyes of diabetics and fellow healthy AMD eyes respectively. In putatively healthy eyes of AMD patients a greater number of hypersensitive amplitude responses were found when surrounding electrodes were considered; however with progression to visible signs of early AMD response amplitudes significantly reduced with no significant difference between occipital and surround electrode responses (−0.109, *p* = 0.181). Further progression to exudative AMD led to significant central sensitivity losses. We did not have and T2D subjects in this cohort who had mild/moderate non-proliferative retinopathy to determine if similar results are replicated. However other mfPOP studies from our lab have demonstrated sensitivity loss and response delays with progression to mild/moderate NPDR^25^. That study explicitly compared the diagnostic power of regions of sensitivity loss, hypersensitive regions, and the asymmetry between regions. For T2D eyes normal fundus appearance mfPOP sensitivity losses performed at chance level (0.50), hypersensitivity at AUC values around 0.70, and asymmetries at up to 0.88. Note that asymmetry adds the effects of hyper- and hypo-sensitivity across congruent regions in the two eyes. Asymmetries provided perfect performance (AUC = 1.0) for eyes with mild to moderate retinopathy^25^.

**Figure 4:**
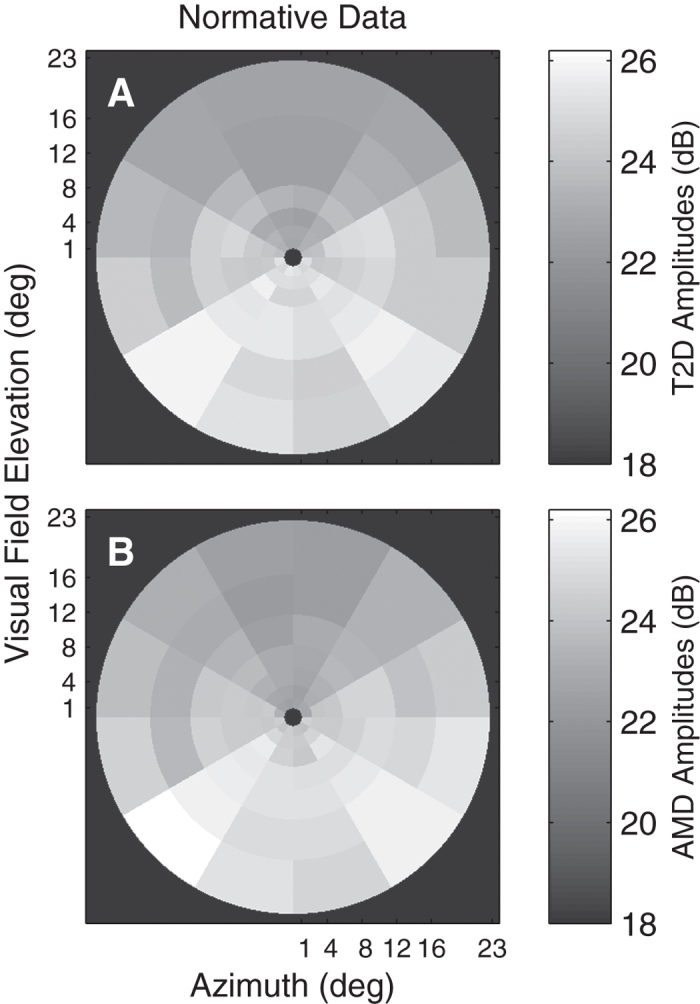
Mean response amplitudes for control subjects in AMD and T2D. A and B show the means across the Occipital Pole electrode data for the normal control eyes, where the input for each region was the peak RMS value from each electrode for each region. Data are presented as if for left eyes with the temporal field at left. (**A)** Normative data for the 44 normal control eyes of the T2D study. (**B)** The normative data from the 56 normal control eyes of the AMD study. The minimum amplitude in either plot is 22.3 dB. The black background corresponds to 18 dB allowing small differences it be seen across the field. The T2D and AMD data were collected 4 months apart and there were no common subjects, nevertheless they are remarkably similar. The gradient of responses from superior to inferior is typical for VEPs for scalp electrodes.

The method for calculating hypersensitivities for mfVEPs (and mfPOP) is quite simple. One first requires normative data as illustrated by Fig. 4 for whatever measure one is interested in. If the subject numbers are not large and the ages are not too diverse the median across control subjects at each region is recommended. Alternatively a linear model of the mean response of control subjects at each region with effects for age and sex could be computed, as was done for Fig. 4. The differences between the data at every visual field region of every subject and the median or model normative data are then computed and the sign of the deviations noted.

**Figure 5:**
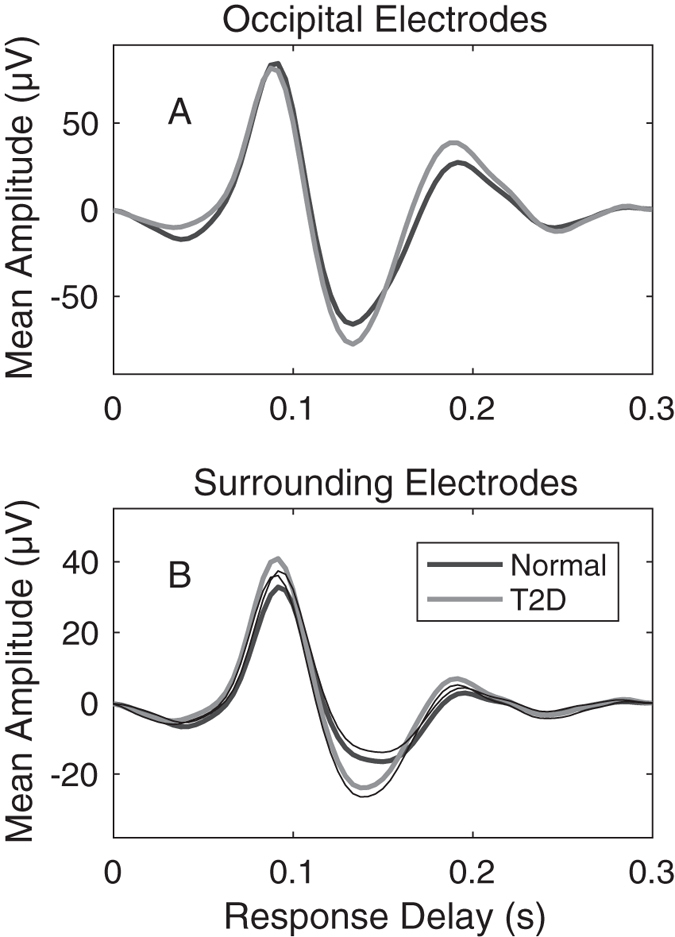
Mean waveforms computed across eyes, subjects, visual field regions (n = 84) for the two electrode arrays from the T2D study. (**A**) Occipital pole electrodes (n = 12) for normal subjects (black waveform) and T2D patients with no retinopathy. Each trace is the average of 2 × 23 × 84 × 9 = 34,776 measured responses (eyes × subjects × stimulus regions × electrodes). Patient (grey) and control (black) waveforms are very similar. (**B**) Averages for the 2 × 23 × 84 × 8 = 30,912 waveforms from the surround electrodes. On average the surround electrode responses of the patients appear to be larger than those of the control subjects. Quantification of these effects by visual field regions follows. The thin black lines of B indicate the means -1 SE and +1 SE for the T1D and Normal waveforms respectively with correction for multiple comparisons. The lack of overlap would indicate significant difference. Age and sex corrected estimates indicated a difference of the first peak of 0.272 dB at p < 0.001.

**Figure 6:**
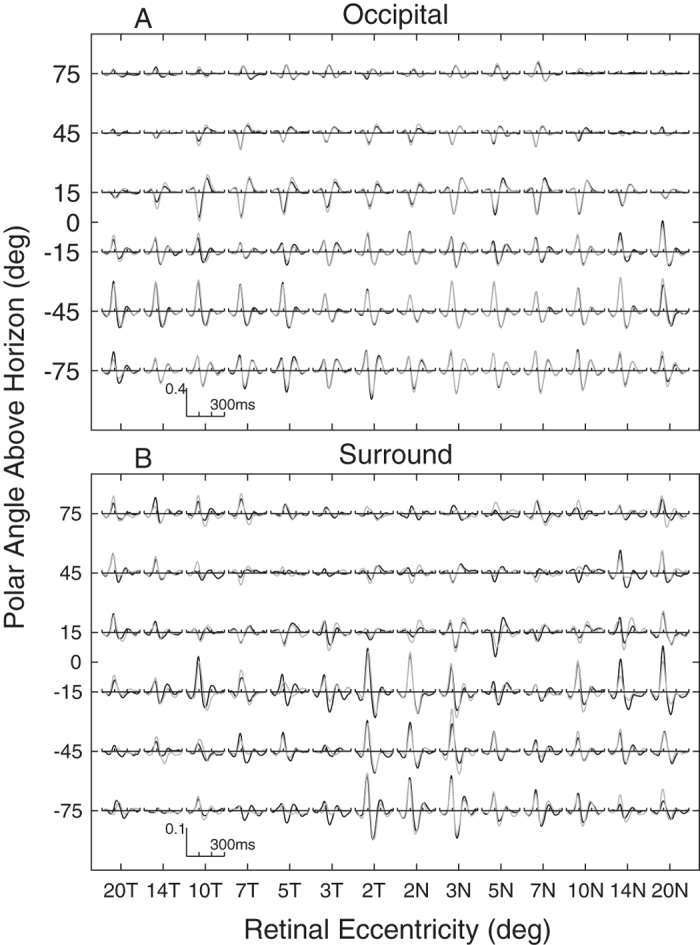
Mean mfVEP waveforms for each of the 84 visual field locations. Mean waveforms were computed across subjects and electrodes, black traces are for left eyes, and grey for right. Where right eye data overlaps left only grey is seen. The data are means across the 51 control subjects from both studies. (**A**) Occipital electrode data show inversion of waveform sign about the horizontal meridian (polar angle = 0 on ordinate). (**B**) Surround electrodes show a mixture near fixation and no inversion peripherally. Eyes were averaged as if both were left eyes with the temporal field on the left. The axes are a log-polar layout as on the cortex, with log-eccentricity from fixation on the abscissa running from 20 degrees temporal (20T) to 20 degrees nasal (20N), and polar angle on the ordinate. The left and right half-rows thus represent the radial spokes of the stimulus array (cf. Fig. 2).

**Figure 7:**
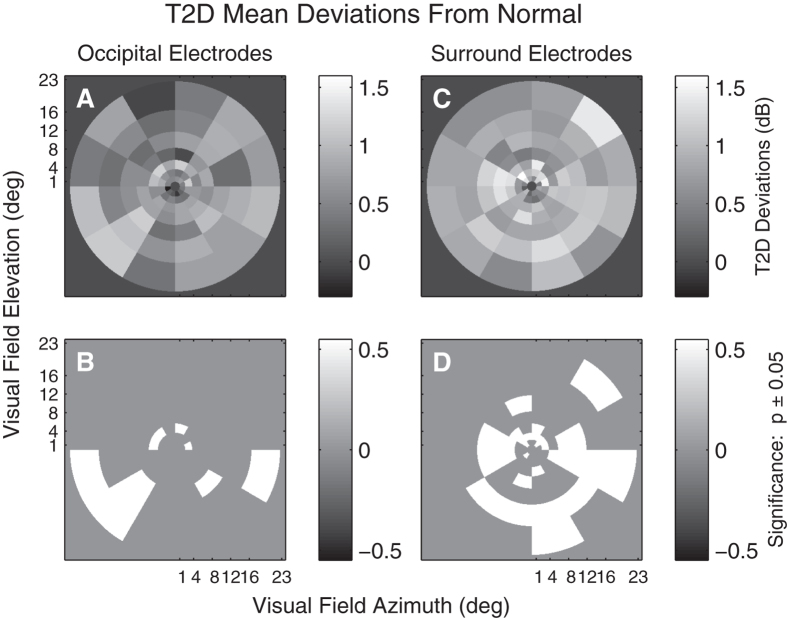
T2D mean response deviations. (**A & C**) Mean deviations from normative data (e.g. Fig. 3) computed across T2D subjects and eyes obtained from a linear model, which also included terms for age relative to 60 years and gender. (**A**) Deviations from normality computed across the Occipital electrodes. (**C**) Deviations from normality computed across the surrounding electrodes. (**B & D)** Indicate those regions deviating significantly from normal (p < 0.05). White regions indicate those which across the 40 subjects and eyes had RMS peak values that were larger than normal. Significant depression of responses would have been indicated in black, but there were no such regions. Consistent with Fig. 4 more regions show significantly elevated responses for the Surrounding electrodes than for the Occipital electrodes.

**Figure 8:**
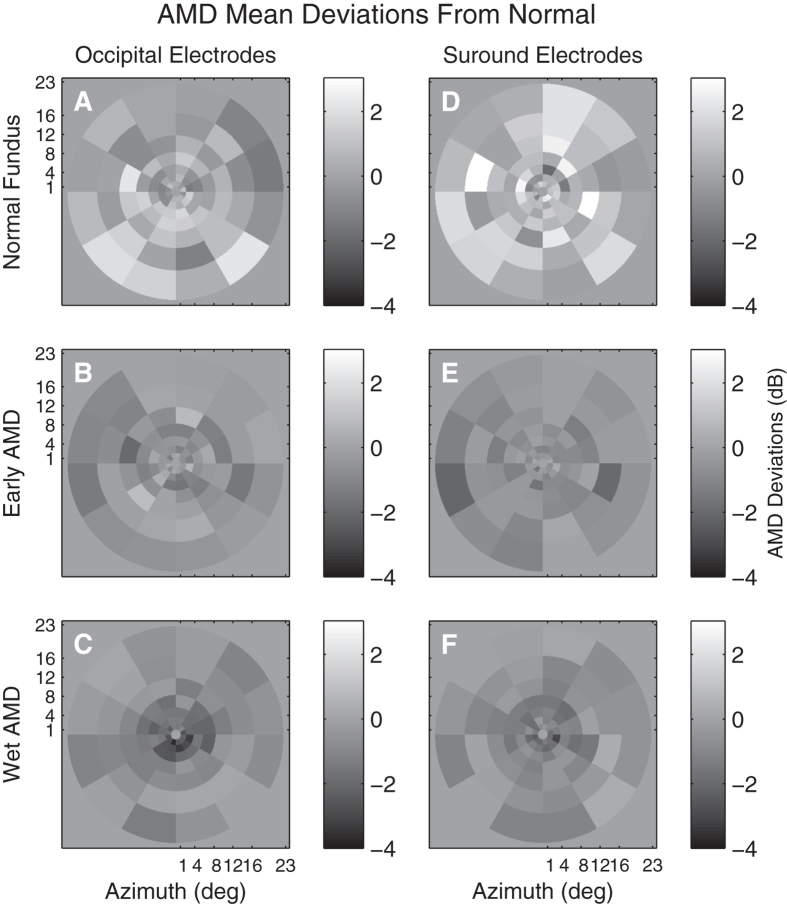
Mean response deviations from the normative data (e.g. Fig. 3B) for three severities of AMD. Response deviations measured from Occipital (**A to C**) or Surrounding (**D to E**) electrodes. The plots are thus similar to Fig. 5A,C. The grey background indicates 0 difference from normal. The top row (**A,D**) are for the 4 fellow eyes that showed a clinically normal fundus appearance for persons aged 74 years. The middle row (**C,E**) are for the 17 eyes with early AMD characterised by drusen and pigmentary changes. The bottom row represent the 17 eyes with exudative AMD (exudative).

**Figure 9:**
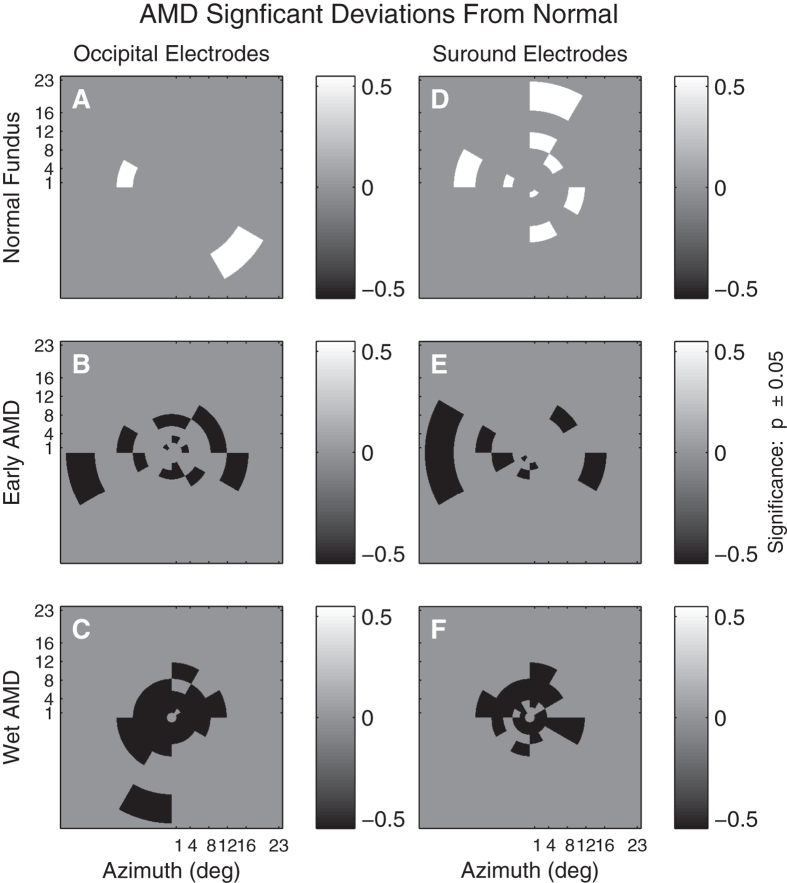
Significant regional visual field deviations across three AMD severities. Visual field regions from Fig. 6 showing significant (p < 0.05) deviations from normal. White regions indicate larger responses than normal, black regions indicate significant response suppression. The exudative AMD eyes (C, F) show marked central loss, while the average losses are more scattered for the Early AMD eyes. Consistent with Fig. 5 the Surrounding electrodes show more regions of response enhancement or fewer regions that are suppressed at peripheral locations.

The issue arises of the reproducibility of the mfPOP method. The T2D study of Figs 5 and 7 included retests of the subjects with mfPOP about 2 weeks after the original test. The mfPOP method of the 2010 study is 3 generations old and so its reproducibility within subjects is not very relevant to current mfPOP practice. We have normative data for our newest methods and have published on the high diagnostic accuracy of that method in early AMD^12^. The 44-region layout of the 2010 method and the latest method are the same, they differ mainly in details of the temporal presentation of the stimuli. The two studies provided data from 23 and 66 subjects in the age range 44 to 62 years. We calculated retest coefficients of variation for each group. For the 2010 and new methods the results were 0.468 and 0.289, i.e. 1.62 times better reproducibility for the newer methods. Further improvements in mfPOP may occur in future. The data from the five studies described in Figs 1, 7, 8, 9 illustrate that the regions of hypersensitivity are also quite consistent across patients, mainly occurring in the periphery in early AMD and T1D.

Our findings suggest that in the early stages of disease before the onset of vascular changes that retinal dysfunction can be expressed at higher cortical centres as hypersensitivity. That outcome is reversed with the onset of structural changes in the retina, where sensitivity loss dominates. Thus, in agreement with standard clinical findings, we found that on progression to exudative AMD both mfPOP (Fig. 1A,D) and mfVEP responses from the central field were significantly reduced (cf. Fig. 9C and F). Overall our results suggest that mfVEP and mfPOP may identify eyes at risk of progression and possible pathology upstream from the retina.

In addition to hypersensitive mean defects on SWAP perimetry of diabetics^26^, localised hypersensitivities have been reported in SWAP and temporal modulation perimetry in ocular hypertension and early glaucoma^36^. A recent study from our lab also found increases in response deviations averaged across visual field rings in patients with T2D with no retinopathy on both Matrix and Short Wavelength perimetry^37^.

To our knowledge this study is the first to examine occipital and surround electrodes in mfVEPs and test the same cohort with mfPOP on the same day. Previous mfVEP studies in T2D show evidence of amplitude loss and implicit delays in eyes with and without retinopathy, however response delays were most affected^27^,^38^. Wolff *et al*.^27^ have reported a small increase in mfVEP amplitude mean Z-scores for eyes of diabetes patients with no retinopathy, and this decreased with progression to non-proliferative diabetic retinopathy (their Fig. 3).

The interpretation of the larger mfVEP responses from surrounding electrodes is complex and is limited by our understanding of the neural responses of the visual system. First, our results may be representative of retinal cell responses that are upregulated in response to high risk conditions from T2D and AMD. Another factor is that the responses may suggest some compensation driven by the brain, which we have demonstrated using mfVEPs in patients with multiple sclerosis who had not experienced optic neuritis^19^. Further research with mfVEP measures from higher cortical regions is needed to explore potential prognostic functional biomarkers of retinal disease.

The study is limited by the small sample size of the AMD severity categories and the lack of a longitudinal follow-up. In addition we did not examine T2D patients with retinopathy with mfVEPs. Nevertheless, in part becasue mfVEP suffers from long setup times, no study has investigated responses from multiple retinal diseases across multiple objective assessments measured on the same day.

In conclusion, the mfVEP amplitudes in patients with T2D with no retinopathy and healthy fellow eyes of exudative AMD patients are significantly larger compared to eyes that have progressed to end stage AMD. The elevated response observed on surround electrodes may reflect extra-striate input to similar mfPOP responses. Importantly, responses measured from surrounding electrodes were more effected than occipital electrodes and may suggest that mfVEP is identifying preclinical microvascular and inflammatory changes within or upstream from the retina. Hypersensitivity may be valuable in clinical screening of fellow healthy eyes of patients with retinal disease and may serve to identify eyes at higher risk of progression. Further research is needed to monitor progression of fellow healthy eyes of patients with retinal disease in a larger study sample.

## Additional Information

**How to cite this article:** Sabeti, F. *et al*. Comparing multifocal pupillographic objective perimetry (mfPOP) and multifocal visual evoked potentials (mfVEP) in retinal diseases. *Sci. Rep.* **7**, 45847; doi: 10.1038/srep45847 (2017).

**Publisher's note:** Springer Nature remains neutral with regard to jurisdictional claims in published maps and institutional affiliations.

## Figures and Tables

**Table 1:** Demographics of healthy, AMD and subjects with type 2 diabetes.

Subject Characteristics	Healthy	AMD		Diabetic Retinopathy	
		Early AMD	Wet AMD	No NPDR	Mild NPDR
No. of eyes, *n*	86	30	38	42	4
Female (%)	53	70	55	48	50
Age, mean ± SD, y	66.2 ± 5.7	84.8 ± 6.0	77.7 ± 5.4	58.0 ± 6.9	55.5 ± 9.8
Mean BCVA ± SD, BCVA	0.0 ± 0.1	0.3 ± 0.2	0.8 ± 0.6	0.2 ± 0.1	0.2 ± 0.2

SD, standard deviation; BCVA, best corrected visual acuity; AMD, age-related macular degeneration; NPDR, non-proliferative diabetic retinopathy.

**Table 2:** t-test comparisons of mean response amplitude differences between occipital and surround electrodes by study variable.

	Difference of means (dB)	t-stat	*P*
T2D	0.272	5.73	<0.0001
AMD normal fundus	0.532	3.70	0.0003
AMD Early	−0.109	1.34	0.1808
AMD Exudative	0.341	2.81	0.0056
